# Editorial on: Genetic Determinants and Prediction of Antibiotic Resistance Phenotypes in *Helicobacter pylori*

**DOI:** 10.3390/jcm9082469

**Published:** 2020-08-01

**Authors:** Daniela Eletto, Alessandra Tosco, Amalia Porta

**Affiliations:** Department of Pharmacy, University of Salerno, Via Giovanni Paolo II, 132, 84084 Fisciano, Italy; daeletto@unisa.it (D.E.); tosco@unisa.it (A.T.)

The introduction of antibiotics in the treatment of microbial infections represents a turning point in human history. Since then, the average human life increased extensively and infections that could potentially kill patients have become less harmful.

However, the “dream” of antibiotics was soon interrupted by the emergence of resistant strains [1]. The initial events of resistance were observed a few years after the very first antimicrobial molecules (sulfonamides), and continued when penicillin was spread extensively. To control the phenomenon of antibiotic resistance, antimicrobial molecules targeting specifically penicillin-resistant infections were modified, and in 1959, methicillin antibiotics were globally introduced. Nonetheless, the first strains of methicillin-resistant *Staphylococcus aureus* emerged right after one year, suggesting that microbes have a natural adaptability that can hardly be therapeutically limited. This microbial feature is mainly due to the high rate of spontaneous mutations in DNA, which along with the extensive use of antibiotics strongly contribute to the development of antibiotic resistance. Such a phenomenon is becoming a worldwide concern if we consider that over 700,000 people die of antibiotic-resistant infections every year [2]. To counteract this severe spreading of drug resistance, multiple strategies have been developed, such as innovative drugs or new diagnostic tools. 

Lauener et al. [3] focused on drug resistance in *Helicobacter pylori* and on the whole genome sequencing (WGS) as a reliable method to predict antibiotic resistance in *H. pylori* infection. The current detection method relies on phenotypic drug susceptibility testing (DST) which can take up to two-weeks. In such a long timeframe, physicians usually do not wait for the results and end up prescribing broad-spectrum antibiotics, feeding the development of drug resistance. Hence, there is an urgent need for a rapid diagnostic test to quickly identify the most suitable drug.

WGS has recently emerged as an important tool for surveillance and antibiotic resistance control, because of advances in sequencing technologies, decreased pricing, faster analysis times and reduced operator handling [4,5]. Though the WGS is currently mainly used in academic or surveillance projects, it can potentially become part of routine diagnostics. The integration of WGS into diagnostics still requires several advances in both parts of the laboratory workflow: the ‘wet’ laboratory part (extraction, library preparation, sequencing), as well as the ‘dry’ bioinformatics part. 

Lauener and coworkers, in their study, compared phenotypic DST results with the predictions based on the presence of genetic determinants identified in the *H. pylori* genome by WGS. They found high congruence between DST and WGS results, as they associated single nucleotide polymorphisms (SNPs) in target genes with drug resistance. In particular, they showed a clear correlation between the occurrence of point mutations in the 23S rRNA, *gyrA*, and *rpoB* genes of *H. pylori* and macrolide, fluoroquinolone, and rifamycin resistance, respectively. As stated by the authors, WGS could provide a more complete picture of resistance determinants, since new polymorphisms, potentially conferring drug resistance, cannot be detected by targeted molecular assays. 

Although the work provides interesting data about the potential application of WGS as a diagnostic tool to predict specific drug resistance, it leaves unsolved points. For example, they were not able to correlate metronidazole resistance to mutations in *rdxA* and *frxA* genes, likely because there is not a general consensus about metronidazole resistance and the relative gene mutations. 

The real advantage of WGS as a diagnostic tool for resistance prediction consists in the possibility to analyze clinical samples without time consuming subculture procedures. Although genomic technologies are rapidly evolving, the whole procedure still requires the implementation of extraction techniques, especially for *H. pylori* infected samples because of low bacterial DNA amounts obtained from gastric biopsies. In this regard, there is encouraging evidence from virology showing that amplification and sequencing techniques have almost fully replaced virus culture. Therefore, we believe that it is only a matter of time before this will also occur for bacterial infections.
